# Homicidal Monomania
*"Examen Médico-Legal des Faits relatifs au Procès Criminel de Jobard." Par J. Artaud, D.M., Médecin en Chef des Aliénés de l'Antiquaille. Paris et Lyon: 1852.


**Published:** 1852-10-01

**Authors:** 


THE JOURNAL

OF

PSYCHOLOGICAL MEDICINE

AND

MENTAL PATHOLOGY.

OCTOBER 1, 1852.

V

### Art. I.—HOMICIDAL MONOMANIA.*

When any great criminal, rendered *great*, in the common acceptation of the term, by the enormity of his delinquency, is arraigned before the tribunal of justice, the bench, the bar, and all the avenues of the court are packed with a dense mass of spectators, belonging to all classes of society, who will endure any amount of personal inconvenience rather than not see the notorious criminal, whose trial, however shocking its details, they will persevere in listening to with intense interest. We can imagine the scene before us. The verdict of "guilty of wilful murder" has been returned, the sentence of death solemnly pronounced, and, when the fatal morning arrives, a rush is made to the scene of execution; the space surrounding the scaffold is crammed almost to suffocation; there is a sea of moving heads and uplifted ghastly faces—such as the Opium Eater describes in one of his most agonizing dreams—every eye being fixed with intent and savage earnestness on the machinery of the cumbrous drop. Streams of human beings may be observed extending far away through all the adjacent streets and alleys; the windows of every house, up to the attic storeys, are filled; the roofs above them dangerously crowded; and every ridge of wall or gable-end upon which a footing can be secured, is taken possession of to catch even the most distant glimpse of this revolting spectacle. A hasty observer might ascribe all this apparently morbid curiosity to the principle laid down by Hobbes, that man possesses naturally a cruel and ferocious disposition; but the more reflecting philosopher will discover that other causes are in operation, less derogatory to human nature. How

* "Examen Médico-Legal des Faits relatifs au Procès Criminel de Jobard." Par J. Artaud, D.M., Médecin en Chef des Aliénés de l'Antiquaille. Paris et Lyon: 1852.

are we to account for the gratification which people derive from witnessing the performance of a deep tragedy? The explanation appears to us obvious. The desire of indulging in emotions of pity is, as Dugald Stewart has shown, one of the active principles of the human mind. There is a conscious pleasure in the anticipation of this excitement; hence persons of sensibility enjoy, even through the haze of tears, pathetic situations on the stage. They provide themselves with handkerchiefs, knowing they will weep with unaffected sincerity, and not as Juvenal describes the hired mourners at a Roman funeral, who "with well-feigned tears their eyes they taught to weep." The same principle induces many persons to witness an execution. But, without dwelling on the causes which have in all ages, and among the most enlightened nations, congregated together such immense masses of people to witness these cruel exhibitions, let us concentrate our attention upon the poor wretch who stands trembling with fear on the brink of eternity, for, as in one of Rembrandt's awe-inspiring conceptions, amidst the gloomy concourse of the multitude around him the whole light of the picture falls upon that solitary figure, surrounded as he is by the ministers of justice, who are themselves doomed to execute the sentence of the land. Upon this miserable object let us, we repeat, fix our attention, and endeavour, as psychologists, to trace the causes, whether physical or moral, which have urged the mind through so many guilty phases; for we may rest assured that vices are transitional, and increase in their enormity as the turpitude of the one becomes absorbed in the turpitude of the other; hence no man ever, on a sudden, became thoroughly abandoned: *Nemo repentè fuit turpissimus.* There are gradations in crime—successive stages of mental perversion and moral degradation. The problem therefore which we propose to ourselves is one of deep and perplexing interest: its solution affects the pure administration of justice, and the welfare of every community.

The accomplished Madame de Stael, in one of those brilliant essays in which she so often and unexpectedly brings into dazzling and fascinating light some of the most recondite truths in philosophy, observes that "man is complete in every individual man." In other words—or rather to follow up this observation—the type of one individual mind is common to all mankind. There are certain fixed and permanent principles of action which are universal, and govern the whole human race. The law which Geoffroy St. Hilaire pointed out as visibly determining the uniformity and unity of organization throughout the animal creation, is not restricted merely to physical structure, but governs also our mental and moral constitution; hence the same faculties, the same feelings, the same sympathies, are in constant operation. The mind of a Bacon or a Newton, a Shakspeare or a Milton, represents the type which must ever characterize the mind of every great philosopher or great poet;—with different degrees of success, they must cultivate the same faculties. And so also is it with our moral nature. The good who are immortalized by their virtues bear in the features of their character a certain resemblance toeach ot her: they are like stars at different altitudes illumining the same heaven. The evil-disposed, on the other hand, preserve the same type of wickedness, varying only in degrees, according as their crimes may reflect intenser and darker shadows on the face of humanity: hence Domitian, Caligula, Nero, do not stand alone; they have their prototypes and rivals in the eastern and in the western world—the Ali Pachas and Zingis Khans, the Robespierres and Dantons, and other monsters as execrable, for the type remains constant. With other criminals more vulgar than were any of the twelve Cæsars it is the same: "Man is complete in every individual man." Upon this principle therefore we are justified in selecting any conspicuous criminal for the purpose of analyzing the state of mind—whether in health or in disease—which existed when he perpetrated the offence, and the particular motives, as far as they can be discovered, by which he was actuated.

Without entering into any profound psychological disquisition respecting the principles of human action, it will, we apprehend, be at once admitted that all our actions originate from causes acting from without or from within. They are objective or subjective. A man exposes himself to temptation; he listens to and hears the plan laid down for a highway robbery or for committing murder, and he calculates the amount of booty and the advantages he is to obtain. All this is external, and purely objective. The reflections, however, to which the proposal gives rise, originate in the mind itself, and suggest a series of impressions which are purely subjective. The desire of gain, or many other motives, soon begin to tamper with the understanding, and obscure the judgment; reason, no longer under its own educational control, then misapplies its divine attributes, and lends its powers to assist in the consummation of the crime devised: hence objective causes give rise to a long series of subjective mental operations. In such cases the intellectual faculties may be in their normal or healthy state, and deliberately reject the remonstrances and admonitions of conscience; there are, however, other cases in which the mind itself, participating visibly in defective or diseased cerebral organization, is obviously weak and incapable of any subjective power of resistance. We may, indeed, satisfy ourselves on this point, by comparing the heads of criminals upon a large scale with the heads of lunatics—the interior of a gaol with the interior of a public asylum. Every civilized nation has allowed the existence of insanity to be received as an exculpatory plea in criminal cases; and although our courts of justice too frequently present the unseemly exhibition of expert counsel endeavouring to puzzle witnesses, and perplex the evidence, by quibbling with physicians unaccustomed to the tactics of cross-examination, for the purpose of obtaining a verdict in favour of the side upon which they are retained,—still the broad fact is admitted, that the state of the criminal's mind is to be taken into consideration, and his responsibility or irresponsibility determined by medico-psychological evidence. "The judgment of the court," says Hoff'bauer, "ought to be governed by the opinion of the physician; and no physician should be permitted to give evidence who has not made a speciality of the study of mental diseases." In a tragedy by a favourite modern poet, one of the judges, in passing sentence on a state prisoner, is made to observe significantly, "We try the crime, the motive heaven will judge;" and many a victim, really insane, has been so sacrificed; but a more benign spirit pervades the principles of Christian legislation. The crime *alone* is not to be tried in the abstract; but all the circumstances attendant upon its perpetration are to be taken into consideration. We gather from the highest legal authorities, that death upon the scaffold—the *ultimum supplicium*—is not intended to avenge the outraged laws of humanity, but to deter others, by example, from committing the like offence. "The execution of an offender," says Sir Edward Coke, "is for example," *ut pœna ad paucos, metus ad omnes perveniat;* and he adds, that the execution of a madman would be a "miserable spectacle," and one of "extreme inhumanity and cruelty," because it could not be regarded as an example to others, the irresponsibility of lunatics being universally recognised.

Unfortunately, however, insanity assumes so many forms, and presents itself under such a variety of aspects, that the plea is, we admit, often very disputable. It is upon these occasions, we conceive, that the *opinion* of the medico-psychologist, founded upon facts which accord with his observation and experience, should be permitted to weigh as evidence; for in all other cases of medical jurisprudence, affecting the cause of death by violence or accident, the *opinion* of the profession is held to be sufficiently valid to be received for the guidance of the jury; but in adjudicating upon the existence or non-existence of insanity in criminal cases, medico-psychologists are constantly desired by the court to adhere to *facts,* and not to give opinions, as if, indeed, the opinion of a medical man, derived from observation and experience, were not the interpretation of a fact as he beheld it; for, after all, it is the opinion he forms of symptoms which can alone guide him in the treatment of every disease, whether it be bodily or mental. The truth is, that all people, in the weakness of humanity, fancy themselves capable of pronouncing judgment upon each other's motives and actions; and when cases of great medico-legal interest come before our public courts, non-professional men, who may be excellent lawyers, but who have not investigated the pathology of the mind in connexion with the functions of the brain and nervous system, and who have had no experience in insanity, fancy themselves as capable of drawing a proper diagnosis, and pronouncing a valid opinion, as men who have for many years devoted themselves to the study and practice of this department of the profession. We believe the time is not far distant when the progress of medico-psychology will vindicate the authority of medical men who make insanity a special study. In the meantime, obscure and doubtful cases will necessarily continue to provoke vague and irrelevant discussions. To return: it must be acknowledged that in many cases there is a very great difficulty in drawing the line of demarcation between sanity and insanity, nor are the rules laid down in the text-books upon medical jurisprudence by any means satisfactory. Nosological definitions are useful in enabling a student to methodize his study of diseases incident to the body; but in actual practice these distinctions are soon lost sight of: it is the same with the generally accepted classifications of mental disease. There is, however, one form of insanity which must be familiar to every practical observer--viz., monomania, which comprehends that form of disease in which the mind is under some specific delusion, or some one morbid train of ideas, which no power of reason can rectify. Some recent authors upon insanity have ventured to doubt, upon very insufficient grounds, the existence of monomania; because, they contend, the order and succession of the morbid phenomena implicate generally the aberration not of one, but of many of the mental faculties. This is purely a psychological error; for, as Sir William Hamilton has remarked, "it should ever be remembered that the various mental energies are only possible in and through each other; and our psychological analyses do not suppose any real distinction of the operations which we discriminate by different names. Thought and volition can no more be exerted apart, than the sides and angles of a square can exist separately from each other." We cannot disentangle and separate the intellectual faculties as we might the threads in a skein of silk; thus, the operations of imagination blend almost with every form of thought and feeling; hence Wolfe observes, "*in omnem actum perceptiones influit imaginatio.*" We do not, in speaking of monomania, refer to the aberration of a single faculty, but to the fact of the mind, with all its faculties collectively, being absorbed in a false impression upon one subject, while upon other subjects it is capable of thinking and reasoning correctly. The celebrated visionary Swedenborg, who was constantly in a state of ecstatic mania, fulfilled the duties of his office so well that the king of Sweden ennobled him. The late estimable Dr. Prichard states, in his work on Insanity, that he knew a doctor in laws who had taken it into his head that all the freemasons had entered into a league against him; yet in other respects he was perfectly sane, and held with credit a chair in an university. We will grant that the mind of such persons, if carefully watched, may be found in some instances to be not altogether in perfect health; but the predominant illusions being confined to one train of associations, the prefix *μονος* to the substantive term *mania* appears to us peculiarly well chosen to designate this disease. Shall we deny that a man who is under the delusion that he is the emperor of the world (such a patient we now have under our care), yet who can talk coherently on other subjects, is labouring under monomania? There is, perhaps, not an asylum in the kingdom which has not within its walls some patient or patients who are deranged upon a given subject, yet rational upon all other topics, and perfectly capable of entering into the amenities and participating in the pleasures of society. "The word monomania," says Esquirol, "involves neither a system nor a theory; it is the expression simply of a fact observed by the physicians of all ages." It often happens, observes Daquin, that a person who is very intelligent and sensible upon other subjects, is as much deranged on some particular point as any of those patients who, being declared lunatics, are confined in asylums. He adds that intense and long-continued reflection may produce some violent and sudden impression on the brain, which may give rise to erratic ideas which become indelibly fixed upon the mind. When, therefore, we use the term monomania, we do not contend that the mind may not be weak and vacillating on many subjects, but simply that this form of insanity is characterized by the predominance and persistency of one particular delusion, which may affect either the intellect or the moral feelings, or implicate the derangement of both. And here it is important to observe that we cannot take a philosophical view of any of these cases without bearing in mind that the disorder may consist either in a derangement of the intellectual or the moral faculties, that is to say, a clear distinction must be drawn between intellectual insanity and what Dr. Prichard has designated moral insanity—the "*manie sans délire*" of Pinel, the "*manie raisonnante*" of Esquirol. We are indebted to Dr. Prichard for having, in his treatise on Insanity, very clearly identified and established the existence of moral, independent of intellectual insanity. In such cases there is a morbid perversion of the feelings, affections, and active powers, without any illusion or erroneous conviction being impressed on the understanding. There is no disturbance of the intellectual faculties, which, nevertheless, cannot control the moral conduct. Cases of this description constantly occur.

About two years ago, without being provided with the usual forms of admission, a gentleman presented himself at a lunatic asylum in the metropolitan district, and begged that he might be received as a patient. He stated that he had just left his solicitor, from whom he in fact brought a letter of introduction, confirming his account of himself, and that it was necessary he should be placed under some form of restraint, for he had an irresistible desire to murder his wife or one of his children. He then added, that the preceding day he was walking in his garden, when he saw his wife and little girl approaching towards him. His eye at the same moment caught the sight of a hatchet lying on the gravel walk, and he described that he had the greatest struggle within himself to escape out of the garden before he seized it to strike, perhaps fatally, one or other of them. He loved his wife and child, he affirmed, dearly; but the homicidal idea haunted him continually, and he felt that he could not trust himself alone in their presence. It should be added, that the last night he slept at home he did attempt, in the middle of the night, to strangle his wife, and would have succeeded had not her cries in the scuffle brought in timely assistance. In the midst of all this, during the explanation he gave of his case, he expressed himself well and rationally. His intellect appeared to be unclouded, and it turned out that he was at the same time in communication with his solicitor respecting some proceedings in the Court of Chancery, upon which he gave perfectly sane instructions. After some necessary delay, in order to procure the forms prescribed by the act of parliament, he was admitted into the asylum; but always when his wife or child came to see him, he required an attendant to be present at their interview. Here we may remark that Esquirol repeatedly declared his conviction that there exists a species of homicidal madness, in which "no disorder of the intellect can be discovered." The murderer is driven, as he describes, by an irresistible power; he is under an influence which he cannot overcome—a blind impulse without reason. It is impossible to divine the motive which induces him, without interest or disorder of the intellect, to commit acts so atrocious and so contrary to the laws of nature. On the criminal side of Bethlem, and in the county asylums where homicidal lunatics are confined, many such cases will be found. We were told by Hatfield, who lately died in Bethlem, that when he went to the theatre prepared to shoot George III., he perceived and reasoned clearly enough upon all that passed around him. The idea which possessed him was, that if he could kill the king, the Messiah would immediately appear on earth, and the reign of the millennium begin; and, in illustration of his self-possession, he mentioned that, when standing at the pit-door expecting it to open, the people around pressed and crowded inconveniently upon him, when a young woman, putting her hand upon his shoulder, said, "Sir, you are hurting me; the handle of your umbrella is running into my bosom." "I could not," he added, "help smiling at the time; for the handle of what she supposed my umbrella was the handle of my pistol, which I held concealed within my coat, under my arm."

We were some time ago consulted by the friends of a gentleman, who was a highly-educated and accomplished man, and a very agreeable companion. We could not in conversation detect any aberration of the intellectual faculties; but he had a propensity to set fire to various descriptions of property (*pyromania*). He had set fire to his own premises, and had contrived afterwards to set fire to three different lunatic asylums in which he was successively confined, but fortunately neither of them was destroyed. He was, as a matter of course, in the house to which he was next sent, closely watched; but after his death, it was found that he had managed to secrete a number of lucifer matches within the lining of his hat. It is well known that many persons have an irresistible propensity to steal, and, without any assignable motives, will commit petty thefts in the houses of their friends, and in public shops. Such cases frequently come before the public. This morbid propensity is exhibited by many declared lunatics, who will steal and accumulate in different parts of their dress all sorts of articles—nightcaps, handkerchiefs, forks, spoons, tobacco pipes, rags, bits of iron, half-picked bones, portions of bread, cheese, &c. We knew a patient who was permitted, in a lunatic asylum, to indulge harmlessly this fancy. When stripped of this miscellaneous gear, he appeared a thin and lathy figure; but presently his clothes would begin to expand around him, and he would increase in size until it became necessary to unpick the lining of his coat, waistcoat, breeches, and relieve him of his imaginary booty. We have also heard the case of a poor woman, who, after wandering about in the open country in a state of lunacy, was sent to an asylum, where she for a long time persisted in refusing food, unless she could steal it; her meals were therefore put in an out-of-the-way corner, on purpose for her to take her food clandestinely, which she habitually did, under the idea she was stealing it. Suicidal mania is another variety of this form of disease; it is known to be hereditary, and to prevail at certain seasons of the year. Sometimes, also, it is epidemic. This morbid propensity—which overcomes the strongest of our natural instincts, that of self-preservation—frequently co-exists with a perfectly healthy state of all the intellectual faculties.

Among those cases of moral insanity which most frequently demand legal adjudication, and which always excite great public interest, are those of homicidal monomania—men arraigned for murder, who were the victims of this morbid propensity, which they could not control or resist.

In the case of the gentleman above referred to, there was no aberration of the powers of reasoning; his judgment was unimpaired, and he was, excepting under this impulse, habitually self-collected. The moral perversion of his nature nevertheless required to be controlled, and if possible corrected. The opinion of Dr. Prichard is, that all cases of monomania begin with a disordered state of the feelings and inclinations, and end in producing intellectual insanity. We know a gentleman subjected to restraint, and there are many similar cases under similar circumstances, in whom this morbid propensity is very strongly developed; he is habitually restless and agitated; and if he cannot kill one person, he would fain kill another. When asked by what motive he is actuated, he evades the question. "Has the person you threaten ever done you wrong? have you any feeling of hatred against him?" "No," he will answer, "not the least; but he must die!" in saying which he will make a gesture, as if assassinating or striking him; yet does this person not labour under any delusion or hallucination to which the suggestion can be ascribed. In the same asylum is a female patient, in humble circumstances of life, who constantly implores that her hands may be fastened behind her, lest she should attack, for the purpose of killing, persons around her, or destroy herself. The whole conversation of this woman is homicidal and suicidal; she has attempted self-destruction; she constantly says she knows and feels she is shaken in her mind, but no specific delusion or hallucination of any kind appears to exist.

One of the most remarkable and interesting cases illustrating this form of insanity, homicidal mania without aberration of intellect, recently occurred at Lyons; the medico-psychological evidence has been collected and published, with observations by Artaud, whose work has suggested the previous observations. Anthony Emmanuel Jobard was born on the 4th of February, 1831, in the village of Essertenne, Haute Saône; his parents were respectable; and his mother and younger sister are described to have been very religious. The infancy of Jobard was passed at home, without the occurrence of any remarkable incident; at twelve years of age he was admitted to communion in the Catholic Church; and the priest of Essertenne declared that no child ever gave him greater satisfaction. His habits were regular, his disposition gentle, and he was very social and kind to his companions, with whom he was a great favourite. Having received his first communion, he attended mass regularly, assisted in the offices, and was punctual in the discharge of his religious duties; nevertheless, at this early period of life he addicted himself to a degrading and solitary vice. At thirteen years of age he left his native village, and with letters of introduction from his late pastor, he was sent, to complete his religious education, to M. D'Oligny, the canon of Dijon, and here remained at school for three years; leaving the house only to go to the lodgings at which he slept. Here also he is reported to have given his masters every satisfaction; but whatever may have been his exemplary conduct outwardly, at the expiration of this time,—when, be it observed, he was only sixteen years of age,—in addition to the vice to which we have adverted, he began to frequent the society of disreputable women. His career of vice began therefore at a very early age, and causes were brought into operation which notoriously pervert the moral feelings, and undermine the strength of the intellect. We are no apologists for crime. The young Jobard had already entered the seductive labyrinth of temptation, and, doubtless, gave unbridled indulgence to his passions: albeit, in the presence of his preceptors he maintained the appearance of being a virtuous and well-conducted youth. The good canon at Dijon was already interested in his welfare; and being now sixteen years of age, and old enough to prepare himself for the active business of life, under his recommendation he was received as a clerk in the establishment of M. Thiebaut, a highly respectable clothier and draper at Dijon. This mercantile firm, which had been long established, bore a high character; the persons employed in it were boarded and lodged in the house, and their conduct, as a matter of course, subjected to the general surveillance of the heads of the family, it being understood that any one who committed any infraction of the rules of religion and morality would be liable to summary dismissal. Upon these terms Jobard was admitted, and received, independent of his board and lodging, in the beginning 200 francs a year, which sum was gradually raised until it reached 450 francs. His conduct continued to all appearance good: he was kind, affectionate, and sober; fond of playing with the children of the family; and the care he took of a pet lamb obtained for him the title of "*Père nourricier de l'agneau*," a circumstance indicating elements and feelings of humanity which should not be lost sight of in the sequel. Here also he was constant and punctual in the discharge of his religious duties; he assisted in the service of the church on Sundays, attended the sermons which were delivered at Christmas, confessed and took the sacrament at Easter; all which, it is stated, he did without any apparent ostentation. There can be no doubt, however, that he still privately—when absent from the house of his employers—persisted in the same immoral habits, which he carried to an inordinate excess, and, at the same time, blended his misconduct with religious, or we should rather say, superstitious feelings, often reasoning upon scripture passages, and addressing exhortations of reformation to his reprobate companions. He would advise the very females with whom he associated to give up their vicious course of life, and on one occasion took very great pains and interest in endeavouring to recommend one of these unfortunate women to retire into a convent, and there expiate by penitence the errors of her past career. This may appear strange, but the contradictions in human nature are very marvellous. The notorious Burke, who was hanged for murder in Scotland, which he committed for the purpose of selling the remains of his unfortunate victims to the anatomical schools, was very partial and kind to children. He preached religious sermons, and the whole series of his murders was suggested by his confederate Hare reading aloud one winter evening the death of Ben-hadad by Hazael, in the second book of Kings.* Incompatible as true religion must ever be with every description of crime, yet we frequently find the almost inarticulate voice of conscience reminding the worst natures of its mandates. Once deeply and at an early age implanted in the mind, true religious and moral principles cannot easily be uprooted, but spread like pure ore through a corrupt soil, blending with elements it fails to purify. We do not accuse such men of hypocrisy; they express in such moments feelings which have become a part of their moral constitution, and which can never be thoroughly eradicated.

These habits of criminal self-indulgence, commenced, as we have seen, so early in life, soon began to undermine the health of Jobard, whose nervous organization, while yet a youth, could not fail to become thereby enfeebled, and otherwise affected. The health and spirits he had enjoyed in the little village of Essertenne, soon failed him at Dijon; he suffered frequently from intense headache, attended with a sense of weight upon the brain, giddiness, and a general feeling of bewilderment; which distressing symptoms ended in a copious bleeding from the nose, which doubtless relieved the cerebral congestion. On one of these occasions the attack assumed a very alarming character, as may be inferred from the following account given by Dr. Noirot, a physician at Dijon, who, upon the occasion of the trial to which we must presently refer, made the following deposition, in the form of a certificate or affidavit. We translate it literally: "I, the undersigned, Doctor of Medicine of the faculty of Paris, certify as follows :—A few years ago, the precise date of which I can scarcely determine, but which,

* This is a very curious fact. The diabolical suggestion arose from Hare reading the account given (verse 15, chap. viii.) of the death of Benhadad, who was thus killed by Hazael: "And it came to pass on the morrow, that he took a thick cloth, and dipped it in water, and spread it on his face, so that he died." Burke and Hare adopted the same plan. They made their victims drunk, and then covered the mouth and nostrils with wet cloths. Sometimes, by kneeling on the epigastrium they forced a deep expiration, which emptied the lungs, and the wet cloths prevented the re-admission of the air. This murderous method was so physiologically scientific, that it was suspected to have been suggested by some anatomist. This was not true; the above statement came out in evidence.

from different circumstances, I believe to have been in the winter of 1844-5, I was called one evening by Madame Perle, a "*sage femme*" at Dijon, to attend a young man who was lodging in her house, Ant. Em. Jobard. They informed me, upon visiting him, that he had for many days previously complained of severe headache, and that he became on that morning delirious. He was so when I saw him; and from this fact, the state of his pulse, and other symptoms, I apprehended inflammation of the membranes of the brain (*meningitis*). Accordingly I proposed that he should be immediately bled; he obstinately, however, resisted the operation, and I thereupon ordered the application of leeches, and then withdrew. The following morning, I found the application of the leeches had been neglected; but during the night a very copious nasal hæmorrhage had taken place, which salutary crisis cut short the disease. He no longer complained of the sense of weight and pain in the head, accompanied by general weakness, which he had endured during the whole of the previous week. I heard no more of the patient, and lost sight of him up to the present period. (Signed) L. NOIROT, D.M.P.; and dated Dijon, 7th November, 1851."

We have already referred to the opinion of Dr. Prichard, that moral insanity frequently terminates in intellectual insanity, the disordered state of the feelings eventually affecting the faculties of the mind and the brain. Professor Heinroth goes further than this; he contends that moral depravity is the essential cause of insanity. Violent passions, sinful indulgences, want of mental discipline, upon this theory give a preponderance so impetuous to all the evil tendencies of our nature as to completely destroy the power of self-control, and subvert the understanding. The case of Jobard would appear to go far in supporting the theory of the learned professor; indeed, there can be no doubt that extreme indulgence in vicious propensities, habitual intemperance, and depraved habits generally, will produce disease of the brain and nervous system; the predisposition, however, to this self-abandonment may be regarded as a disease in itself, and will be frequently found to be hereditary. To proceed, however, with the history of Jobard. On the 12th of September, 1851, he left the house of his employers, not premeditating the tragedy in which he was about to perform so conspicuous a part. His cousin had commissioned him to buy some windows for a church; and remitted him 50 francs, which was the maximum price he was to give.

On Sunday, the 14th of September, he attended mass, and afterwards vespers, as usual, and after the latter service, went to a restaurant's with three of his fellow-clerks, belonging to M. Thiebaut's establishment, and there they dined together very cheerfully, nor did either of them drink to excess. When the dinner was over, Jobard suddenly rose from the table, and left his companions, following hurriedly into the street a German singing girl, with whom it appears he went home. He remained with her about ten minutes, and then returned to the café, when he called for a glass of *kirsh;* his expression of countenance was anxious and haggard, and the waiter noticed that he was much agitated. Immediately afterwards he took up his cane and hat, and without saying a word to his companions, who were still at the table, he again sallied out, and went in search of a cutler's shop, for the purpose of purchasing a knife; but it was after nine o'clock, and the shops were then shut. Foiled in this intention, he directed his steps to a house of ill fame in the Rue Quentin, where he passed the night. His conduct there, which we abstain from describing, was exceedingly extravagant—apparently insane. About two o'clock in the morning, he hastily dressed himself, said he must be off by the railway to Paris, and, without uttering a word, left the house; and without being prepared for the journey, proceeded to the railway station, and asked for the first train to Paris. He was informed it would not be there until seven o'clock. The train, however, for Châlon, came up while he was talking, and he instantly took his ticket, and proceeded *en route* for Lyons. Arrived at Châlon, he took an omnibus which conveyed passengers to the steam-boat which leaves the pier of that town for Lyons. We must now premise that we are tracking the steps of an assassin—the question of homicidal monomania will be presently considered. In the train from Dijon to Châlon, he tells us that he could not explain the nature of his feelings; he could not think or reflect on anything—(*j'avais la tête vide;*) he ate a little, however, on the way. Arrived at Lyons, he felt much fatigued, and walked mechanically to a restaurateur's, where he dined. He drank half a bottle of wine, but ate very little. During dinner, he asked the waiter to direct him to a cutler's. The man did so, but Jobard could not find the house; he therefore got into a cabriolet, and desired to be driven to a shop where they sold knives, where he bought a knife, with as much coolness as if it had been a lead pencil, or any other harmless article. This done, he sought out and entered another house of ill fame in the Rue de la Cage, determined, he afterwards stated, to kill one of the female inmates. He was introduced to a girl named Rachel; he remained with her half an hour, but she was so pretty that his arm was arrested—he could not strike the blow—and was tempted to delay for a few hours his resolution; he therefore left her, promising to return to her at night after the theatre, and he adds, that if he had done so, he would have stabbed her in her sleep, (*je lui aurais percé le cœur pendant son sommeil.*) This resolution, he confesses, he did not take without some qualms of apprehension, for he was afraid that before he could effect his escape he would be torn to pieces by the exasperated women of the house.

This happened on Monday, the 15th of September, 1851. Upon arriving in the town, he stopped as he walked along the streets to read a bill of the play, and he determined to go to the Théâtre des Celestins. Upon leaving this house, therefore, after walking for about ten minutes in the Jardin des Plants, he went to a café opposite the theatre, and there waited until the doors opened. He then paid for his admission and took his seat in the gallery, *aux premières,* but at the end of the first act of the second piece, which was entitled *Adrienne Lecouvreur,* he changed his place, and went into the amphitheatre, where he sat down behind a pillar. He there saw at a little distance from him a little girl about ten years of age; he grasped his knife, and would have killed her, but she was beyond his reach, and he could not move towards her without attracting attention. Another little girl, apparently between twelve and fifteen, sat nearer and a little to his right, but she too was beyond his grasp. His attention was next directed to a lady who sat immediately before him; she wore a gray silk dress, and as he stood up and looked down upon her, he saw a portion of her breast uncovered; but at this moment he heard steps behind him, and looking round saw the manager of the theatre, whom he knew personally, and who had just entered the house by a door near him. He instantly pretended to be cleaning the nails of his fingers with the knife; turning towards him, he smiled, and after exchanging a few gracious words with each other, the manager passed on. Suddenly, a scream, sharp and piercing, resounded through the house, and persons were seen rising confusedly around the place whence it proceeded. The fatal deed was perpetrated. With deadly aim and force, he had plunged the knife into the bosom of the unfortunate lady, and as she uttered that thrilling shriek, she withdrew with her own hand the knife from the wound, and, covered with blood, fainted in the arms of the persons near her. Her husband, who was sitting next her, not having any idea of the fatality of the blow, turned round upon the assassin, and exclaimed, "What have we done to you, that you should thus strike my wife?" "Nothing!" answered the imperturbable Jobard; "you have done nothing!" He stood calmly with his arms crossed upon his breast, and added—"I am a miserable being; do what you please with me; it is not my wish to escape!" He was immediately arrested; he did not make the slightest resistance, and as he was being conducted to the Hôtel de Ville, he said, "I am well content," (*Je suis satisfait actuellement*). In the meantime, the poor lady was removed into the "*foyer*," (the green-room of the theatre;) the wound now appeared evidently mortal, and in about five minutes she expired. She was a young woman, the daughter of M. Chabert, the *Proviseur* of the Lyceum at Limoges, and the wife of M. Ricard, the Professor of Mathematics in the same institution. Melancholy to add, she was in the family-way—*enceinte de six mois.* As he struck her from behind, she never even saw the person of her murderer.

The excitement and consternation produced by so tragical an event, very naturally spread through the whole city. Who was the assassin? By what motives was he actuated? An eye for an eye—a tooth for a tooth—blood for blood—was the instinctive outcry of popular fury; and if he could, like the wretched parricide, Damiens, have been torn to pieces by the force of horses driven furiously in opposite directions, to which his limbs were attached, and had had melted lead poured into his eyes and ears and veins, the multitude, exasperated at the fate of so innocent a victim, would not have bestowed the slightest sympathy upon his agonies. But we live in a Christian age; the criminal code of enlightened nations no longer sentences the infliction of torture; for justice must ever now be tempered with that divine mercy which becomes

> "The thronèd monarch better than his crown,
> * * * *
> And is the attribute of God himself."

There are, it is true, some persons, and we have met with several, who contend that, sane or insane, every man who takes away human life should himself be put to death; which summary judgment would cut the Gordian knot of every medico-legal difficulty; but, in accordance with the milder dispensations of Christian jurisprudence, it behoves us to take into consideration all the exculpatory and extenuating circumstances which might have existed. We agree with Herr Heinroth, that in such a case as the above, sin and madness would appear to be identical, for who can determine the exact point at which the power of self-control may cease to influence human actions? True, many criminals are hurried onwards by an impulse which they cannot repress; but who can determine the resisting powers of motives which must escape analysis? How can we decide, within the secret sphere of another man's mind, where his responsibility ends and his irresponsibility begins? We would repudiate entertaining or pandering to notions of false humanity; the poor wretch we have described upon the scaffold trembling on the brink of eternity may have deserved his fate, and we will not in that case join the sympathies of the unthinking crowd of spectators; but when the existence of insanity is clearly proved, and its symptoms have been as unequivocally manifested as those which characterize any physical disease incident to the human body, then we hold that the plea of insanity ought to be unequivocally admitted. How does it happen that crime appears to be so often hereditary, independent of the force of example, unless there were a predisposition in the human mind to succumb to its evil influences? The members of one particular family will, through a whole generation, be found to have been addicted to incurable drunkenness—those of another, successively imprisoned or transported for theft or burglary. There can be no doubt that insanity is hereditary; and so are the vices of successive generations; but it deserves to be noticed particularly, that insanity, in its transmission, will assume different forms. The monomania of one man may be on religion; his next of kin may have the disease under the form of suicidal or homicidal mania: which is the case with one of the patients above referred to, who has a near relation at the Agapemone, who believes in the inspiration of Mr. Prince, and the divine ordinance of playing at "blind hockey."

To proceed with the history of Jobard. He was conducted a prisoner to the Hôtel de Ville, and placed in one of the dungeons, where his first care was to take the sleeves off his paletot, to prevent them being soiled, and then he fell on his knees in prayer. He conducted himself during his examination before the "Juge d'Instruction" with great calmness; his countenance maintained its usual expression, and his pulse was regular, and between 65 and 70 in the minute. When informed of the death of Madame Ricard, he said, "Dead, is she?—so much the better; since I wish them to put me to death." He then asked if there had been time for her to see a priest before dying, and upon being answered in the affirmative, and informed that he had prayed for her, he expressed his satisfaction, and added, "I am sorry I was obliged to put her to death; I pity her and her family, and in that sense I regret it; I shall repent before God; as for me, I cannot make myself better understood than by saying,—I regret nothing." In his first examinations he persisted in the same tone, confessing but not regretting the act; on the contrary, he said, "I always knew, even when I contemplated it, that it was a crime for which I was responsible before God and man." He confesses, "I bought the knife as coolly as I would a crayon, or anything else. I struck her as I would have done a block of wood" (*"je la frappai comme j'aurais frappé un morceau de bois"*). At another time he said, "I committed it without reflection. If I had reflected properly—if I had confided in any one—if they had made me reflect upon it—I should have desisted." The murderous weapon was produced before him; he looked upon it with indifference. The distressed husband was confronted with him, and he evinced very great emotion. He was then conducted into the presence of the dead body, and when he beheld his victim, he appeared on a sudden horrified, became extremely agitated, and his pulse rose, gave 88 pulsations in the minute, and was thready and intermitting. He appeared unable to support himself, but nevertheless he almost immediately recovered his self-possession, and spoke with his usual calmness. Upon leaving the room he again appeared much affected; bewailed the fate of his victim, and the distress of her family, but for himself, he again added "*I regret nothing.*" The following day he was again examined at the Palais de Justice, and now, he altogether altered his tone; "If my time," he exclaimed, "were this day to come over again, I would not commit the deed I have done; yesterday I endeavoured to render my condemnation inevitable;—to-day I would desire to live; if that were permitted me (he continued, with apparent emotion), I would seek the *frère* Manuel, and lay my heart open to him as I now do to you; my repentance would then, I think, be complete; I cannot yet sufficiently explain my conduct, but I feel myself already so far changed." Since his imprisonment, it should be observed that he had experienced nasal hemorrhages, which may have relieved the brain, and conduced to the return of a more natural state of feeling. He now appeared to be more afraid of death than he had ever before been; he continued restless and agitated, and his sleep was disturbed by frightful dreams; nevertheless, after he had confessed himself, and received absolution, he informed M. D'Artaud "that his mind had regained its composure." "Do you think," he was then asked, "that after having premeditated and committed so atrocious a crime, confessing it, and receiving absolution, is a sufficient reparation?" "Yes," answered Jobard, "*lorsque j'aurai terminé ma pénitence!*"

We have hitherto not alluded to one very striking feature in the character of Jobard—viz., his consummate hypocrisy. We have seen him while yet a boy preserving outwardly the most exemplary and virtuous conduct, yet indulging at the same time secretly in the most vicious habits; hurrying, after assisting at mass and at vespers, from the church to the brothel; and when we read the confessions which he made before the magistrates, and particularly in the letters he addressed to his family, we shudder at the expressions connected with religion which are interwoven, as if to palliate his wickedness. He tell us that the hypocritical life he had led weighed (as well it might) upon his conscience; he determined, therefore, to become really religious, and endeavour to ensure his salvation. "It was impossible for me," he writes to his sister, "to expect salvation from the conduct I pursued; I had not force of resolution enough to change my mode of life, and I therefore said to myself, if I once kill a person, I should repent, I would do penance, and God, who is good, would forgive me." One of his first resolves, then, he pretends was to assassinate a priest as he was leaving mass; his soul, he tells us, would then have mounted directly into heaven, and he would have prayed and interceded for the forgiveness of his assassin. He then proposed to enter upon a soldier's life, with the idea of being killed in action, and so ridding himself of his burthensome life; but his mother, we are assured, dissuaded him from this project. He next thought of selecting for the sacrifice he meditated, some infant, the soul of which, never having committed sin, would enhance its powers of intercession on his behalf. Once also, it is said, that he thought of joining in a conspiracy to assassinate the President of the French Republic, and meet his death as a regicide. The idea of committing suicide also occurred to him, but a pious horror of self-murder restrained his hand from the poisoned cup or dagger; so he ascribes to his sense of religion the merit of having resisted this evil suggestion. We candidly confess, however, that we place no reliance upon these different confessions, which bear upon their face the evidence of being, as far as his motives are concerned, tissues of contradictions. The apology for his crime, the idea *"j'ai tué pour être tué,"* never peeps out until after his arrest; and upon his trial it will be found that he endeavoured to repudiate that explanation in his anxiety to make the act appear, by his answers to the court, not to have been premeditated. We have, as a general principle, no confidence in the last dying speeches and confessions of criminals, which are concocted in the condemned cell, to justify or palliate their crimes; many of them are suggested by a very natural desire to appear in their last moments to the best advantage, and excite the sympathy of the public; and many, perhaps the greater number of men in such an appalling situation, are so confused and bewildered, that they are unable to give any true or accurate history of the motives which influenced them. We believe Jobard to have been not less a hypocrite after than he was before he perpetrated the assassination; and his whole conduct appears to have evinced a sense only of guilty cowardice. He trembled at the prospect which awaited him; he coquetted with religion and prevaricated with truth every time he opened his lips.

Immediately after the crime was committed—within a few hours—the medico-legal question arose as to the condition of his mind: When he perpetrated the act, was he sane or insane? An ordonnance was issued immediately by the *Juge d'Instruction*, requring Dr. Magaud to examine and report upon his physical and moral state; and Messrs. Tavernier and Gromier, physicians frequently called upon to give medico-psychological evidence before the criminal tribunals, received the same mission. The conclusions arrived at by Dr. Magaud were, that the act was premeditated; the motive alleged in his declaration was insufficient to prove any aberration of the intellectual faculties; that there was a continual conflict between his religious ideas and sensual instincts, which may in some degree have obscured his understanding; but that his sense of moral responsibility, although enfeebled, was nevertheless not destroyed, therefore the evidence was not sufficient to be placed in justification of the crime, although it might be urged in mitigation of the punishment. The report of the Doctors Tavernier and Gromier, in which they also recapitulate all the circumstances of the case, terminated in opposite conclusions. 1st. They were of opinion that at the moment Jobard committed the act he was in a state of dementia (*monomania homocido-suicido*). 2nd. That Jobard could not be considered responsible for an act which he committed without the participation of his will. 3rd. That as this description of insanity leads to disastrous consequences, society should exercise its right of placing Jobard in a situation to prevent him committing any other outrage, and that he should be confined for life in a lunatic asylum.

The result, therefore, or rather the opinions expressed in both of these reports, are thus far approximative; the one recognises enfeeblement (*affaiblissement*), the other derangement of the moral sense. The one pleads for mitigation of punishment, the other recommends deprivation of liberty, and confinement, for life, in a lunatic asylum. The view taken by Dr. Arthaud, who has been at the pains of collecting the whole evidence in a very circumstantial manner—not, indeed, omitting the most trivial circumstances that could be interpreted in his favour, is, that Jobard, when he committed the act, was insane—and that his insanity was characterized by a general disturbance of the intellectual faculties; in support of which opinion he refers to the fact, that insanity, in different forms, had declared itself both on the paternal and maternal side of his family. His grandfather, at the age of fifty or sixty, upon the loss of an action at law, was for several months insane. His disease assumed the form of lypemania. He continually cried "I am a lost man," believing he was about to be arrested; at length, after obstinately refusing food for a fortnight, he died of inanition. His grandfather's brother also died in a melancholic state, and the cases were very analogous. His cousin Rosalie Jobard, when twenty years of age, had an attack of acute mania. She was confined in the lunatic asylum of Maréville between two and three years, when she was discharged cured. On the maternal side, one uncle became insane at an advanced age, exhibited symptoms of lypemania, and died of senile dementia. Another was affected by melancholia, would associate with no one, ate the roots of vegetables as he pulled them out of the ground, and wild fruits, and threatened to injure those who approached him. Another uncle, in a state of ordinary insanity, was confined in the lunatic asylum at Besançon, where he died. Again. One cousin was an epileptic—and another, imbecile from birth; Dr. Arthaud traces these and some other examples in the family of Jobard of mania, lypemania, dementia, epileptic mania, and idiocy. It is impossible, he then adds, that the hereditary transmission of insanity can be doubted.

Pinel, Esquirol, Marc, Falret, Descuret, Baillarger, Moreau, and all modern writers on insanity, consider hereditary transmission one of the most ordinary predisposing causes of this deplorable malady. According to Esquirol, one-sixth of the cases of this disease which occur among the poor, may be attributed to hereditary transmission, and the proportion, he adds, will be found still higher among the higher classes. With him, I am disposed (says Dr. Arthaud) to think this calculation is below the actual proportion found. Physicians who are indeed specially connected with asylums, know how difficult it is to arrive at any exact statistics on this point. M. Michéa goes further. He states we must agree in admitting that at least one-half, not to say three-fourths of such patients have had, or still have, some members insane in their family. By Marc, also, hereditary transmission is considered to be the most predominating of all the causes of insanity; "it plays," he observes, "so important a part in the production of this disease, that whenever its existence can be proved in a medico-judicial inquiry, it is almost sufficient to establish the reality of some lesion of the understanding," (p. 118.) We are not, be it observed, disposed to doubt the moral of insanity of Jobard, and that his vicious habits undermined the healthy functions of the brain and nervous system, and impaired generally his intellectual faculties; but sufficient powers of mind still remained, which he might, we suspect, in the beginning have controlled. The false appearances under which he contrived to disguise, before his preceptors and employers, the real features of his character, and his punctual attention to the outward forms and ordonnances of religion, may be referred to in support of this opinion. He was sincere enough, doubtless, in his devotions, so long as the excitement which prompted them, under the influence of his imagination, lasted; but when this intellectual effort was over, he then gave unbridled sway to all his worst passions. "*Quand je priais,*" said he, "*je priais comme un saint; un instant après, le vice m'entrainait, et je me laissais aller sans résistance possible.*" We have dwelt emphatically upon his self-acknowledged and habitual hypocrisy, and have furthermore expressed a general disbelief in the statements he made at different times, explanatory of the motives which actuated him in his guilty career. This description of deception, it should be remembered, is one of the most prominent characteristics of moral insanity. Under this form of disease, a young man will endeavour to appear exceedingly circumspect in his outward conduct, while, at the same time, his perverted feelings prompt him to commit all sort of delinquencies; he will invent mischievous stories respecting persons he has no particular enmity to; he will tell the most abominable falsehoods in apparently the most truthful manner, and is always prepared with some plausible reason and ingenious fabrication to account for any misconduct which he may have committed.

On the 23rd of March, 1852, Jobard was arraigned before the Assize Court of the department of the Rhone for the murder of Madame Ricard. Upon examining the body, it was found that the knife had penetrated between the second and third ribs of the left side, traversed the anterior part of the corresponding lung, and piercing the left ventricle of the heart, the point of the weapon terminated in the interventricular wall of the right ventricle. The facts of the case were too evident and incontestable to admit of any doubt, therefore the medico-legal depositions, which were endorsed at the back of the indictment, respecting the state of the prisoner's mind, constituted the principal evidence. During the trial, the prisoner maintained his self-possession, but was manifestly anxious respecting its result, and endeavoured, by the answers he gave to the interrogatories put to him, particularly to remove the idea that the act was premeditated. He was ably defended by M. Dubost, his advocate, and after the President of the Court had carefully summed up the evidence, Antony Emmanuel Jobard was declared guilty of having voluntarily, and with premeditation, committed a homicide on the person of Joséphin Annaïs Chabert, the wife of Ricard, whereupon he was condemned to hard labour for life—"*aux travaux forcés à perpétuité.*"

We do not impugn the justice of this verdict, or consider that it was an aggravated sentence; because the homicidal act was clearly neither instinctive nor impulsive, but the result of premeditation and pre-arrangement, although the particular victim had not been selected for the assassination until a few instants before the act was committed. Furthermore, we fully admit that Jobard was morally insane; but there was not such an amount of intellectual aberration as to obscure or pervert the understanding; on the contrary, he himself acknowledged that he was conscious he was about to perpetrate a crime, and he knew that he would be responsible for the deed before both God and man. Hence we recognise—in the derangement of his moral or affective faculties and propensities only—attenuating circumstances which very properly suggested to the jury the recommendation to mercy; but we do not see sufficient evidence to justify the full exculpatory plea of insanity. There can be no doubt that a person so dangerous ought to be deprived of his liberty, and, provided the hard labour which he is condemned to perform be not incompatible with health, or likely to shorten the duration of life, we would raise no objection also to this part of the sentence. Indeed, we often, in visiting the criminal wards of Bethlem, St. Luke's, and some of our public county asylums, have been struck at observing so many strong and healthy-looking criminal lunatics sauntering, with their hands in their pockets, idly about the corridors and courts of the building: how much better would it be for these men to be obliged to work at some compulsory labour—some occupation adapted to their particular capacities and previous conditions in life. Assuredly it would be beneficial to their bodily and mental state, and conduce materially to their recovery: for whether they are destined to remain under *surveillance* or not, their restoration to sanity is to themselves, and all around them, of equal importance. The man who may, under some delusion consequent upon cerebral disease, have attempted even the life of his sovereign, may recover his reasoning powers, and it is manifestly important that he should do so before his death; wherefore he is entitled to receive as much care and attention as any other patient who, upon recovery, becomes entitled to his liberty.

To return to the point from which we started. We commenced the present article by describing a spectacle which is too frequently exhibited in our densely crowded metropolis. Whilst the multitude assembles round the scaffold to witness an execution, from an epidemic feeling of excitement which gives rise to motives they do not themselves understand, the medico-psychologist is, we have stated, called upon to view the object of popular curiosity with very different feelings. It is his duty to ascertain, as clearly as he can, by the history of the case and his own observations, the state of mind which may have led to the perpetration of the act for which the criminal has been condemned. In a state of health, as we have premised, "man is complete in every individual man:" it is the same in disease—one disorder, bodily or mental, is the type of the same disorder throughout the world. In a psychological view, the actions of all men may be regarded in a two-fold light; they are suggested either by his moral or affective faculties—his feelings, desires, passions, and propensities—or they are the result of intellectual motives, originating in the powers of the understanding. The moral may, it is true, blend, and that almost imperceptibly, with his intellectual nature; but it is proved, beyond a doubt pathologically, that the moral and affective faculties may be perverted, and that morbid states of the feelings, and diseases, passions and propensities, may exist without any concomitant affection of the intellect or disease of the brain and nervous system. On the other hand, the intellectual faculties may, individually or collectively, be deranged, and the powers of perception, reason, and judgment so much impaired as to obscure the understanding and lead to the belief in delusions and hallucinations which may suggest very insane actions. The one, as we have explained, constitutes moral, the other intellectual insanity; but they frequently, although originating separately, run into each other and become combined—hence moral often ends in intellectual insanity; but we believe that, *vice versa,* intellectual as often conduces to, and ends in, moral insanity. Applying these remarks to the form of monomania we are now considering, it appears to us that homicidal mania may be subdivided into three varieties:—

1. Moral Homicidal Mania—arising from perversion and a morbid state of the decrees, feelings, passions, and propensities, which the powers of the understanding, reflection, reason, judgment, &c., can neither control nor counteract. Acts of impulsive homicide come within this category.

2. Intellectual Homicidal Mania—arising from some disease of the imagination—some delusion or hallucination—or misapplication and perversion (which often happens in religious monomania) of the reasoning faculties. This variety of the disease is always primarily dependent on some lesion of the understanding.

3. Moral and Intellectual Homicidal Mania—arising from some morbid state of the feelings, passions, and propensities affecting and impairing the controlling influence of the intellectual faculties.

But we have already exceeded our limits; the subject of homicidal mania is extremely important, and we shall ere long again return to it; in the meantime the "*Examen Medico Legal*" relative to the criminal prosecution of Jobard, by Dr. Arthaud, is so valuable an addition to our medico-legal literature, that we recommend it not only to our medical brethren, but to the attention of those members of the legal profession whose practice should interest them in the progressive principles of medical jurisprudence.

